# Association Between Balance and Hip Muscle Strength in Inline Skaters

**DOI:** 10.3390/jfmk10030331

**Published:** 2025-08-29

**Authors:** Lara Sánchez Torres, Iván Nácher Moltó, José A. Navia, Javier Reina Abellán

**Affiliations:** 1 Health Sciences PhD Program, Universidad Católica San Antonio de Murcia, 30107 Murcia, Spain; larasancheztorres19@gmail.com; 2 Physiotherapy Department, Universidad Católica San Antonio de Murcia, 30107 Murcia, Spain; inacher@ucam.edu (I.N.M.); jreina@ucam.edu (J.R.A.); 3 Education Sciences Department, Universidad de Alcalá, 28871 Alcalá de Henares, Spain

**Keywords:** balance, hip abductor, hip adductor, stability, strength

## Abstract

**Background**: Inline skating has rapidly grown in popularity. Early research primarily focused on injury patterns and protective measures. However, its biomechanical similarity to other skating modalities enables the synthesis of existing evidence, emphasizing key physical attributes essential for performance, namely, balance and the strength of the hip adductor and abductor muscles. The interaction between these muscle groups in relation to balance has not yet been examined in inline skaters. This study aimed to investigate the relationship between single-leg static balance and the isometric strength of the hip adductors and abductors, including their strength ratio. **Methods**: A total of 191 amateur inline skaters (aged 18 to 59 years) were evaluated. Balance was assessed through center of pressure displacement using the Footscan^®^ 9 platform, and the maximal isometric strength of the hip adductors and abductors was measured using a handheld dynamometer. A linear regression on the center of pressure (CoP) displacement was performed. **Results**: Age, sex, and skating frequency were the most influential predictors (*p* < 0.001), although strength variables also significantly predicted the CoP (*p* <0.05). **Conclusions**: Superior balance performance was observed in younger individuals, women, and those practicing five or more days a week. Furthermore, single-leg static balance was associated with an equilibrium between adductor/abductor strength, particularly when a low ratio was accompanied by high levels of hip adductor strength.

## 1. Introduction

Inline skating has become one of the fastest-growing recreational sports, gaining global popularity following the development of inline skates [1,2]. It has been adopted not only as a form of aerobic exercise but also as a mode of local transportation and a competitive sport, with its practice increasingly embedded in social activities.

Early research in this field has primarily addressed injury patterns and protective equipment [3], with musculoskeletal injuries reported as the most frequent [1], largely associated with balance loss [4,5]. Muscle strains and tears [6] have also been noted, particularly involving the hip adductor muscles in recreational inline skating, given their role in the recovery phase following propulsion [7].

Inline skating entails significant physical demands—varying by discipline and skill level—including strength, flexibility, aerobic and anaerobic capacity, speed, power, and balance [8,9,10]. Lower limb kinematics and muscle activation patterns are key technical factors in skating performance, as they drive movement [11].

Despite the discipline-specific biomechanical characteristics, notable similarities have been observed between the biomechanics of ice skating and hockey skating and those of individual inline skating movements [12,13]. Research on muscle activation patterns in these sports has shown that concentric contraction of the gluteus maximus plays a major role in power generation [14] during the propulsion phase. This muscle is active during propulsion, facilitating hip extension [15,16], abduction, and external rotation. In contrast, during the recovery phase, eccentric contractions of the hip adductors are responsible for decelerating the stride—a function that has been associated with better performance in skaters [17].

Thus, the hip abductor (ABD) and adductor (ADD) muscle groups are of particular interest due to their essential role in inline skating kinematics. Recent studies in ice hockey have proposed assessing the strength of a muscle group relative to its antagonists—specifically, the hip ADD/ABD strength ratio [18].

Furthermore, inline skating has been described as a sport that challenges postural balance, given the reduced friction area and small base of support required to maintain stability while moving [19]. The term postural control, often used interchangeably with balance [20], refers to the integration of sensory and motor processes that maintain equilibrium. It relies on the interaction of three sensory systems: visual, vestibular, and neuromuscular. The neuromuscular system includes mechanoreceptors located in muscles, tendons, joints, and the skin [21]. Optimal performance of this system is essential for preparing joint movement and achieving functional joint stability [22,23]—both critical for successful skating.

Unilateral dynamic balance is defined as the ability to maintain the center of mass within the base of support during single-leg movements [24], and it represents a fundamental skill for safely and effectively performing many explosive sport actions on one leg [25]. In female athletes, this ability has been positively associated with isometric hip ABD strength, highlighting the abductors’ critical role in maintaining postural control [26].

Hip strength plays a key role in stabilizing posture and balance, and in anatomically and biomechanically connecting the upper and lower extremities [27]. Hip ABD and ADD muscles are critical for frontal plane lower limb stability, influencing pelvic control, femoral kinematics, and dynamic knee valgus—factors strongly implicated in injury risk. Hip ABD function contributes to lateral balance control and has been shown to influence balance performance across the lifespan [28]. Higher ABD strength is inversely correlated with pelvic drop and femoral adduction, thereby enhancing stability.

Conversely, hip ADD, particularly the adductor longus and magnus, along with other internal and external hip rotator muscles, generate substantial forces during high-velocity movements, playing essential roles in propulsion and pelvic stabilization [27,29,30]. These muscle groups act synergistically as a force couple to regulate lower limb mechanics during sport-specific tasks.

Although several studies have examined the influence of hip ABD and ADD strength on balance in different populations, revealing significant correlations [27,28,31,32,33,34], no research to date has evaluated the isolated and relative strength of these two muscle groups in relation to balance in inline skaters. This gap is particularly relevant given the importance of performance optimization and injury risk prevention in a sport with a high incidence of falls and related injuries—reported to account for 88% of recorded cases [5,35,36].

Inline skating requires repeated unilateral stance, lateral push-offs, and sustained frontal plane control. These tasks demand the coordinated action of the hip ABD, ADD, and rotators for stability, alignment, and propulsion. While each group’s role in balance and injury prevention is well-documented, their interaction, specifically the hip ADD/ABD strength ratio, remains unexplored in inline skaters. Understanding this relationship could inform targeted interventions to enhance performance and reduce injury risk in this population.

Based on this context, we hypothesize that the ADD/ABD strength ratio of the hip is associated with single-leg static balance performance in inline skaters.

## 2. Materials and Methods

G*Power [37] recommended a sample above 138 participants to achieve a power (1-β) of 0.95, with α = 0.05 and a medium effect size in a regression test. We gathered 191 amateur inline skaters (105 men and 86 women), aged between 18 and 59 years (*M* = 35, *SD* = 9.32). According to the classification of athletes proposed by McKinney J et al. [38], amateurs are those who perform approximately 150 min of moderate-intensity skating or 75 min of vigorous-intensity skating per week for at least 6 months. Radman et al. [39] concluded that one year of experience (regardless of frequency) was enough to complete a skating skill test.

Inclusion criteria required a minimum of one year of skating experience and regular practice of one to three days per week (d/w). Participants were excluded if they had undergone surgery on the lower limbs within the six months prior to the study, or if they had chronic illnesses or were taking medication that could interfere with physical performance. Most participants were right-footed (82%, *n* = 157) and 18% (34) were left-footed. In terms of experience, 41% (*n* = 79) reported between one and five years, 19% (37) between five and ten years, and 39% (75) more than ten years of experience. Regarding frequency of practice, 60% (*n* = 114) skated between one and three d/w, 27% (52) four or five d/w, and 13% (25) more than five d/w. Additionally, 67% (*n* = 128) reported regularly practicing another sport and 33% (63) only skated.

The study was conducted in accordance with the Declaration of Helsinki and was approved by the Ethics Committee of the Catholic University of San Antonio de Murcia (UCAM) (Reference number: CE052314). It was also registered in ClinicalTrials.gov (ID: NCT05971316). All participants provided written informed consent prior to inclusion.

An observational, descriptive, and analytical study design was employed. Each participant completed an initial questionnaire to collect personal and demographic information, followed by the recording of anthropometric data.

A standardized 8-min warm-up was then conducted, including jogging, joint mobility exercises, and neuromuscular activation routines [31]. Testing procedures were conducted on the dominant leg, in the order described, to minimize the effect of muscular fatigue [40]. Assessments were performed one day before an international skating event (April 2024), which required a minimum skill level for registration. All measurements were carried out in the research classroom of the Faculty of Physiotherapy at the Catholic University of San Antonio de Murcia.

All tests were verbally explained, and participants were allowed one familiarization attempt without data recording. For the assessment of static balance, three 15-s trials were performed, with 30 s of rest between attempts. Average center of pressure (CoP) displacement in millimeters was calculated using data recorded during each repetition with the Footscan^®^ V9 9.5.8 software (RSscan International NV, Paal, Belgium).

Participants stood barefoot on the Footscan^®^ 9 pressure platform, with the second toe of the support leg aligned with the anteroposterior axis of the platform and the medial malleolus aligned with the anteromedial axis. The contralateral leg was flexed at 90° at the knee and held in the air, while the arms were crossed over the chest. The test was performed with eyes open and gaze fixed on a visual target 5 m ahead [8].

Subsequently, the maximum isometric strength of the hip ADD and ABD was assessed. Tests were performed with participants in a supine position on a medical examination table. They were allowed to hold onto the edges of the table for stabilization, and compensatory movements were not permitted.

**Figure 1:**
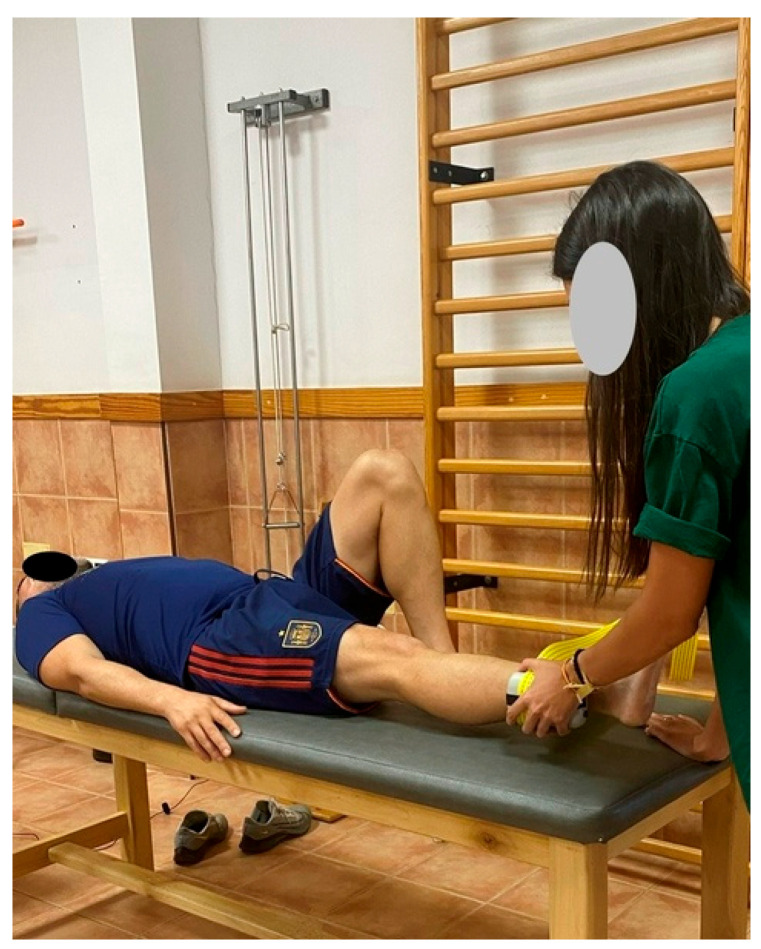
Measurement of maximal isometric hip abductor strength using a handheld dynamometer.

**Figure 2:**
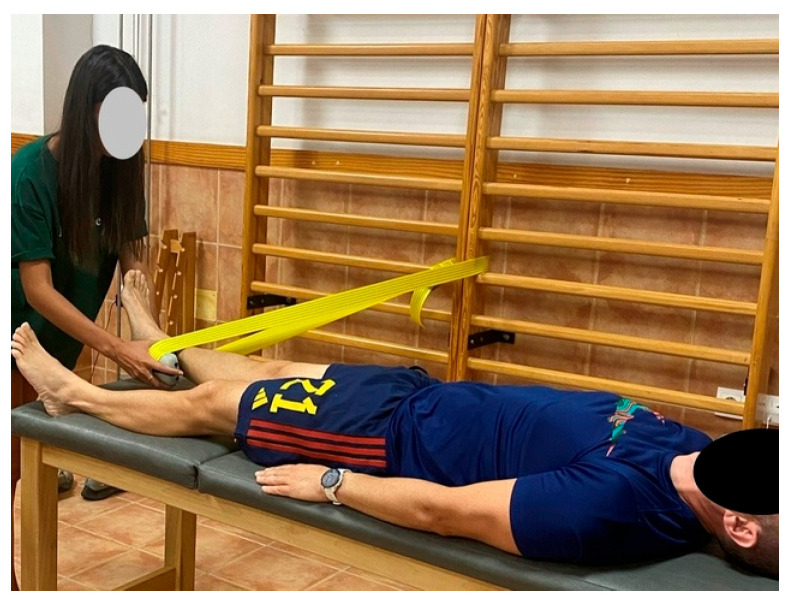
Measurement of maximal isometric hip adductor strength using a handheld dynamometer.

A portable handheld dynamometer (Nicholas Manual Muscle Tester, Lafayette Indiana Instruments, LafayetteIN, USA) was secured with a strap to a fixed vertical structure anchored to the ground. Participants were instructed to perform a 5-s maximal isometric contraction against the dynamometer. The strap was placed 5 cm from the lateral malleolus (for abduction) (Figure 1) and from the medial malleolus (for adduction) (Figure 2). An investigator ensured proper execution, and verbal encouragement was given throughout the test. Four valid trials were collected, and the best result (N) was used for statistical analysis [41,42].

We performed a regression analysis on CoP with the other collected variables as predictors. Assumptions of normality (K-S), collinearity (VIF), autocorrelation (DW), and homoscedasticity (Breusch–Pagan tests and visual inspection of residuals) were verified. IBM SPSS V.29 and jamovi V. 2.6.29 were employed for statistical analysis and figure production.

## 3. Results

Table 1 shows the descriptive measures. The regression model (R^2^ = 0.19) revealed a significant effect of sex and age on the CoP. Adding frequency of practice improved the model up to R^2^ = 0.22, showing better balance among skaters who practice more than 5 d/w in comparison to those who only practice 1–3 d/w. Introducing strength variables (excluding ABD strength due to collinearity) further improved the model up to R^2^ = 0.25.

The final model (Table 2) averaged a VIF = 1.24 and DW = 1.67. Weight, height, or BMI showed no effect on CoP (Supplementary Table S1). Similarly, laterality, years of experience, and regular practice of another sport showed no effect on CoP (all *p*s > 0.05, Supplementary Table S1).

**Figure 3:**
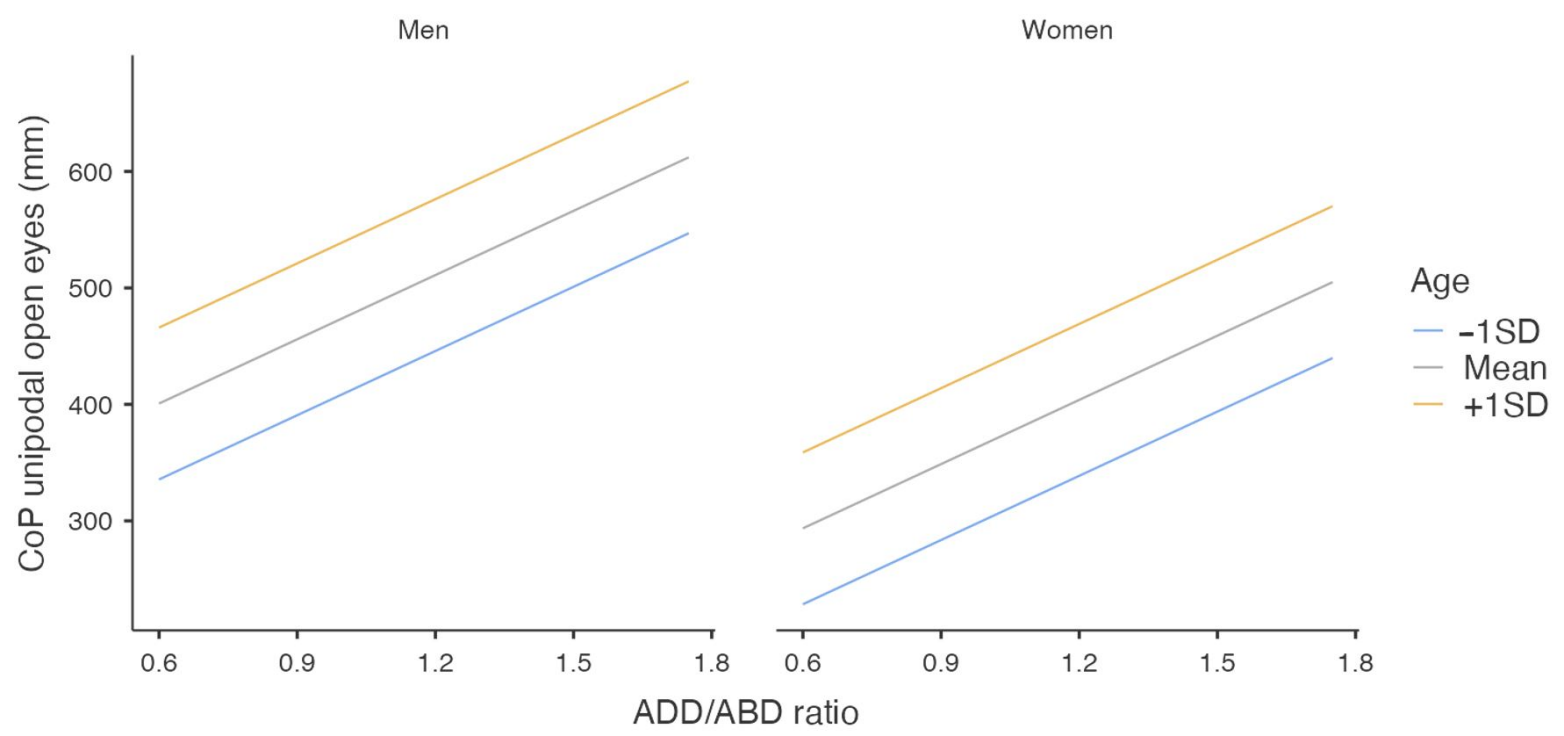
Estimators of unipodal CoP.

In summary, younger participants, women, and more frequent skaters (>5 d/w) demonstrated better unipodal balance. Also, it seems that a lower ratio on ADD/ABD (i.e., more balanced strength) combined with higher ADD strength was associated with reduced CoP displacement (Figure 3).

## 4. Discussion

The main findings of this study indicate that amateur inline skaters with lower hip ADD/ABD strength ratios—reflecting a more balanced strength profile between hip ADD and ABD—exhibited better single-leg static balance performance. In addition, greater hip ADD strength was associated with improved postural control, as reflected by a reduced center of pressure displacement. Finally, balance outcomes were also influenced by participant characteristics, with younger skaters, females, and those who practiced more frequently showing superior performance.

These results suggest that hip muscle balance, particularly the ADD/ABD ratio, may play a role in postural stability in inline skating. This association could have potential implications for both performance enhancement and injury prevention strategies in this population.

Assessing fundamental motor abilities such as hip muscular strength—essential for movement patterns and for maintaining and generating control during single-leg stance phases—and balance capacity could be considered a valuable component of training, as it may help identify functional deficits and provide a basis for both injury prevention and performance enhancement [7,8,9,43,44].

Muscle imbalances between opposing muscle groups are recognized as a critical factor in injury prevention across numerous cyclical sports [45]. Prior studies have identified links between hip strength and balance performance, and several have previously noted associations between balance and hip muscle strength [46,47].

Research on hip muscle activity in ice hockey players offers valuable insights into their contribution to forward skating mechanics. In such athletes, balance heavily depends on the ADD muscles due to the narrow base of support during movement. This necessitates strong involvement of the medio-lateral hip muscles, among others, to maintain postural control in this challenging context [17]. Other investigations, such as those in healthy children, have shown that inline skating training improves both strength and balance capacities [48]. Moreover, interventions focusing on strength and balance training have been shown to enhance skating proficiency [49].

Miller and Bird [50] reported that fatigue in the hip and knee muscles has a greater negative impact on single-leg stability than fatigue in distal lower limb muscles. More recently, Gribble and Hertel [51,52] demonstrated greater postural control deficits following fatigue of the hip ADD and ABD compared to ankle invertors and evertors. These findings support the notion that hip and knee muscles, due to their larger cross-sectional area, generate more force than those acting on the ankle.

Our findings appear to align with previous evidence, suggesting that, in inline skaters, a lower ADD/ABD ratio may be associated with better single-leg static balance. This observation could support exploring the use of this ratio as a potential indicator in future research on performance optimization and injury risk assessment.

The ADD/ABD strength ratio data from the dominant leg provide a new perspective, supporting the idea—proposed by previous authors—that this ratio may serve as a predictor of lower extremity injury risk [18]. These findings may inform future interventions targeting injury prevention or training programs, in the same way that the quadriceps-to-hamstring ratio has been used to evaluate knee and ankle injury risk. Furthermore, better performance on the balance test—reflected by reduced center of pressure displacement—was also associated with higher ADD strength.

While the gluteus medius, a key muscle in forward skating, has received more research attention, its antagonist, the adductor longus, must not exhibit excessive asymmetry in strength or activation. As hypothesized, excessive muscular tension in the adductor region—widely recognized as the most injury-prone area in this sport—may result from repeated eccentric contractions, which act to decelerate the limb during lateral movement [53]. Asymmetries in peak electromyographic activity between the right and left hip ADD muscles (up to 80% reduction in impulse) and altered coactivation patterns with the gluteus medius could increase the vulnerability of the adductor longus to injury [54].

This study also found a strong relationship between static balance, age, sex, and frequency of practice. Younger participants and females performed better on the balance test compared to their older and male counterparts. These findings are consistent with previous research reporting that men exhibit greater center of pressure sway, while women demonstrate better balance regardless of the measurement system or test duration [55]. Male participants generally show greater postural sway, which increases with age. Furthermore, the decline in balance capacity appears to be more pronounced in men, with postural control deterioration beginning relatively early in both sexes (typically between ages 40 and 49), likely due to physiological aging processes and lifestyle-related reductions in physical activity [56,57]. Previous research has shown, in accordance with the results of this research, that in adults, a higher weekly training frequency is associated with greater improvements in muscular strength and balance-related functional outcomes. A positive dose–response relationship has been reported between training frequency and strength gains, with effect sizes increasing progressively from 1 d/w to ≥4 d/w [58]. Moreover, when weekly training volume is held constant, increasing training frequency still appears to offer practical advantages, such as better recovery management and movement quality [59]. Therefore, engaging in recreational sports like in-line skating—which inherently involve balance control and muscular strength—at a higher weekly frequency may result in enhanced neuromuscular adaptations. In this context, frequent skating practice (i.e., more than three d/w) is associated with improved strength and balance capacities, with performance outcomes positively scaling with training frequency.

Despite the large sample of amateur skaters, the study was limited by the short data collection period prior to an international event, which constrained the inclusion of additional participants and variables of interest. As is common in a typical observational design, data were collected at a single time point. Future studies could test and expand upon these findings through experimental designs and by incorporating other relevant variables that may also be related to the observed outcomes.

## 5. Conclusions

Single-leg static balance is associated with lower hip ADD/ABD strength ratios, particularly when accompanied by higher ADD strength. Age, sex, and frequency of practice also influenced performance, with better results observed in younger individuals, females, and more frequent skaters (>5 d/w).

These findings suggest that muscular balance in the hip joint—especially between the ADD and ABD—may contribute to optimal postural control during inline skating. This association could provide useful insights to guide both training and injury prevention strategies in sports requiring high balance demands, while also laying the groundwork for future research in performance diagnostics.

## Figures and Tables

**Table 1:** Mean, confidence interval of the mean at 95%, standard deviation, and range of participants’ measures.

		95% Confidence Interval			
	Mean	Lower	Upper	SD	Range
Descriptives					
**Age**	35.36	34.03	36.69	9.32	41
**Weight (kg)**	68.69	66.79	70.59	13.31	70.00
**Height (m)**	1.71	1.70	1.72	0.09	0.40
**BMI (kg/m^2^)**	23.43	22.94	23.92	3.45	18.62
**CoP (mm)**	416.29	389.37	443.20	188.59	945
**ABD strength (N)**	141.87	137.06	146.69	33.71	169.20
**ADD strength (N)**	150.15	144.01	156.29	43.02	217.30
**Ratio Add/Abd**	1.06	1.03	1.09	0.20	1.14

Note. The CI of the mean assumes sample means follow a t-distribution with N − 1 degrees of freedom.

**Table 2:** Model coefficients on CoP.

Predictor	Estimate	SE	t	*p*
Intercept	138.66	98.49	1.77	0.161
Age	7.00	1.33	4.80	<0.001
Sex	−107.17	33.01	−3.25	<0.001
Days/week				
3–5 vs. 1–3	6.04	28.38	0.21	0.832
>5 vs. 1–3	102.66	38.19	2.69	0.008
Ratio Add/Abd	183.84	72.28	2.54	0.012
Add strength (N)	−0.88	0.43	−2.03	0.043

## Data Availability

Data available on request due to restrictions. The data presented in this study are available on request from the corresponding author due to ethical reasons.

## Supplementary Materials

The following supporting information can be downloaded at: https://www.mdpi.com/article/10.3390/jfmk10030331/s1, Table S1. Regression model introducing all potential predicting variables to estimate unipodal CoP.
