# Supplementary material for: Association Between Balance and Hip Muscle Strength in Inline Skaters
Source: J Funct Morphol Kinesiol. 2025 Aug 29;10(3):331. doi: 10.3390/jfmk10030331 (PMC12452686; doi:10.3390/jfmk10030331)
Supplement: Supplementary file 1 [file jfmk-10-00331-s001.zip › jfmk-3766414-supplementary.pdf]

Supplementary Table S1.

Regression model introducing all potential predicting variables to estimate unipodal CoP

Predictor	Estimate	SE	t	p
Intercept	-217.23	378.59	-0.57	0.567
Age	6.88	1.41	4.87	<.001
Sex (W-M)	-88.06	38.53	-2.29	0.023
Days/week				
3-5 – 1-3	2.96	29.00	0.10	0.919
>5 – 1-3	106.42	38.65	2.75	0.007
Ratio Add/Abd	214.36	76.99	2.78	0.006
Add strength (N)	-1.02	0.46	-2.23	0.027
Heigh (m)	164.47	191.56	0.86	0.392
BMI (Kg/m2)	2.85	4.07	0.70	0.485
Other sport (No-Yes)	-9.10	26.41	-0.34	0.731
Laterality (R-L)	-0.62	32.19	-0.02	0.985
Years of experience				
>5 years – >1 year	31.43	34.37	0.91	0.362
>10 year – >1 year	-24.15	28.10	-0.86	0.391

Note: this full model reached a $R^2 = 0.27$ with an Average VIF = 2.22 and DW = 1.63). It did not significantly ($p = 0.667$) improve the presented model in the paper ($R^2 = 0.25$). Abd strength (originally with $t = 1.12$ and $p = 0.265$) and weight (originally with $t = 0.061$, $p = 0.540$) were removed for collinearity reasons with Ratio and BMI, respectively.
